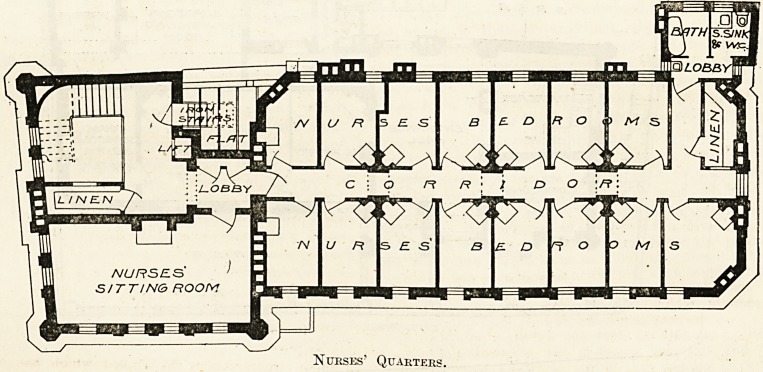# Hospital Construction

**Published:** 1899-05-13

**Authors:** 


### The Institutional Workshop.

### HOSPITAL CONSTRUCTION.

### PADDINGTON GREEN CHILDREN'S HOSPITAL.

In considering the plans of hospitals built within recent years in the more crowded portions of the metropolitan area, one must not tie oneself down too strictly to observing how nearly they approach to the ideal. Especially is this the case in regard to space and separation between buildings, the amount of land available being in many cases so restricted that it is quite impossible to obtain that segregation of ward from ward, and in-patients from out-patients, which one would be glad to see, and which one would expect to find provided in any case in which land was no object. One has not then to judge of the Paddington Green Children's Hospital merely by theoretical standards, but rather by considering how far the architect has shown ingenuity and resource in making the most of the opportunities offered him by the site, and looking at the matter from this point of view there is much in this building which is worthy of great praise. The site is practically a square, a portion of one side of which abuts against other buildings; but only a part of the site is at present utilised for hospital purposes.

The main idea of the full plan is that the two sides of the site, facing Paddington Green and Church Street respectively, shall be occupied each by a block of wards several stories in height, the angle where these two blocks unite containing a staircase common to both. The present portion includes only one of these blocks and the out-patient department, the latter extending quite to the back of the premises, the one-storey building of which it consists covering most of the ground behind the higher block in which the wards are placed. No small ingenuity has been expended in contriving their out-patient department, and by aid of plenty of top lights a fair result has been obtained, although it must be confessed that the accommodation provided is not of the largest. Perhaps it is thought that privacy in dressing and undressing does not matter with children, but those who have witnessed the facility with which one screaming child will demoralise a whole roomful will admit that a better supply of little rooms would have been a great advantage. The wards are very good and well arranged. Perhaps for children it does not

We do not notice any outside emergency staircases for use in case of fire. This, we think, is a serious omission, and one which might have been easily obviated; and while we are on this subject we would point to the position of the lift as being not well chosen. It would appear that the staircase placed at one end is the only means of access to the several floors, not only of the present building, but of the building that is to come. Thus the safety of all the inmates, in case of fire, depends on that one staircase, opening on to the landings of which is a lift running the whole height of the building. Moreover, this lift works in an enclosed lift-

Ward for Boys.

Nurses' Quarters.

matter very greatly, but we notice that the baths are placed against the wall in the bath-room. No doubt want of space has had to do with this also. In fact, the sanitary arrangements are undoubtedly somewhat cramped. The w.c. and the sink are in the same room, and a sink is placed in the ventilating (or cut-off) lobby, a most unsatisfactory arrangement. The same criticism applies to the sanitary arrangements in the top storey, which is devoted to nurses, although in this case the lobby being at the end of a passage the cut-off is more complete. A very pleasant balcony is arranged outside the boys' ward. It is easily accessible, through a lobby, from the ward.

shaft. If an unenclosed lift is made to work up the open well of a wide staircase it will not act as a flue any more than the staircase itself, but an enclosed lift-shaft is a much more dangerous means of distributing suffocating vapour. We think that architects might well turn their attention to outside lifts. They are largely used abroad, they interfere in but the smallest degree with window light, and they not only avoid the dangers inseparable from a shaft, but provide a means of access to the various floors, which, if the machinery is properly constructed, is practically incapable of being interrupted by fire.

## Figures and Tables

**Figure f1:**
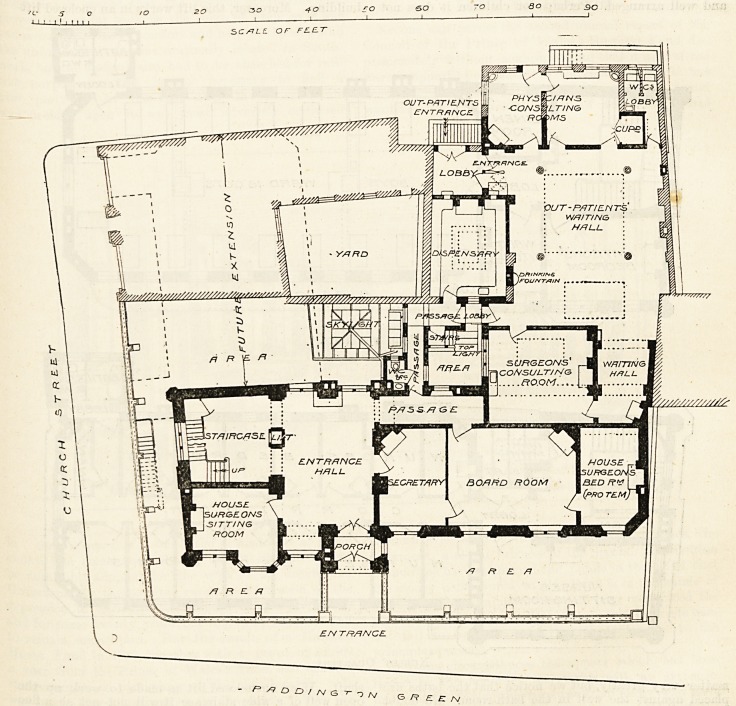


**Figure f2:**
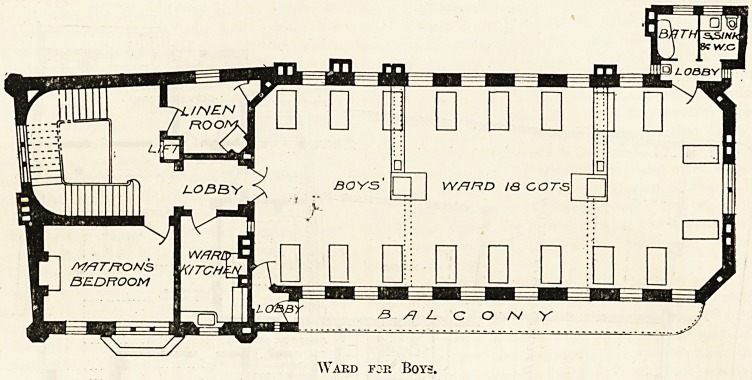


**Figure f3:**